# 1758. Persons who Inject Drugs Leaving Medical Encounters against Medical Advice during an HIV Outbreak in Kanawha County, WV — A Mixed Methods Study

**DOI:** 10.1093/ofid/ofad500.1589

**Published:** 2023-11-27

**Authors:** Adelaide Balenger, Lauren Love Pieczykolan, Robert A Bonacci, R Paul McClung, Christine Agnew-Brune, Stephen Perez, Suzanne Wilson, Alana Hudson, Sherri A Young, Kathryn Curran

**Affiliations:** CDC and Oak Ridge Institute for Science and Education, Atlanta, Georgia; CDC and Oak Ridge Institute for Science and Education, Atlanta, Georgia; CDC and Oak Ridge Institute for Science and Education, Atlanta, Georgia; CDC and Oak Ridge Institute for Science and Education, Atlanta, Georgia; CDC and Oak Ridge Institute for Science and Education, Atlanta, Georgia; CDC and Oak Ridge Institute for Science and Education, Atlanta, Georgia; West Virginia Department of Health and Human Resources, Charleston, West Virginia; West Virginia Department of Health and Human Resources, Charleston, West Virginia; Kanawha-Charleston Health Department, Charleston, West Virginia; Centers for Disease Control and Prevention, Atlanta, Georgia

## Abstract

**Background:**

In 2021, an HIV outbreak investigation among persons who inject drugs (PWID) in Kanawha County, West Virginia identified that patients frequently left medical encounters against medical advice (AMA). Leaving encounters AMA is linked to high rates of readmission and mortality and leads to missed opportunities for HIV testing and HIV care engagement. We identified factors associated with leaving encounters AMA among PWID in this HIV outbreak.

**Methods:**

For 65 PWID with HIV diagnosed during 1/1/2019–6/18/2021 who resided in Kanawha County, we analyzed demographic, clinical, and encounter data abstracted from inpatient and emergency department medical records from one year prior to HIV diagnosis through 6/18/2021. In addition, we analyzed data from qualitative interviews conducted in June 2021 with a purposeful sample of 26 PWID and 45 community partners (service providers, law enforcement, policymakers, and religious leaders), examining themes related to leaving encounters AMA.

**Results:**

We identified 307 inpatient and emergency department encounters, including 80 (26%) encounters during which the patient left care AMA (Table 1). Among 65 PWID with diagnosed HIV infection, 37 (57%) ever left an encounter AMA, and of those, 16 (43%) were not virally suppressed (Table 2). Patients left encounters AMA more frequently when they were diagnosed with injection drug use (IDU)-related infections or when heroin use was documented during the encounter (Table 1). During interviews, providers cited staffing shortages, barriers to implementing integrated care models, and the complex social and medical needs of PWID, as challenges to care delivery among PWID. From the perspective of PWID, barriers to care retention included maltreatment and stigma from clinical staff, social isolation, and withdrawal symptoms.

**Figure:**
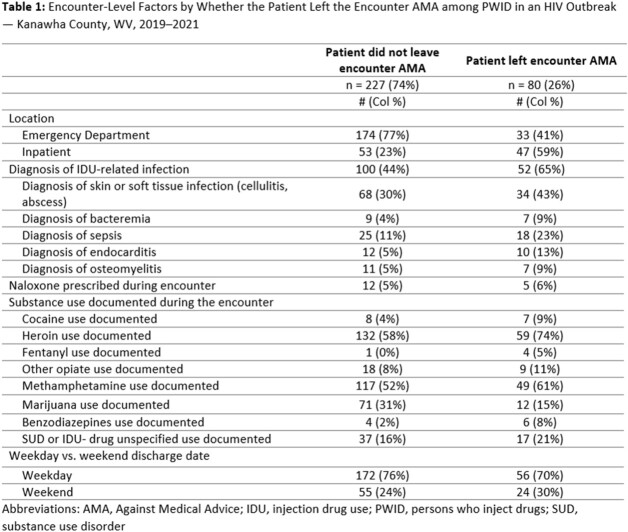

**Figure:**
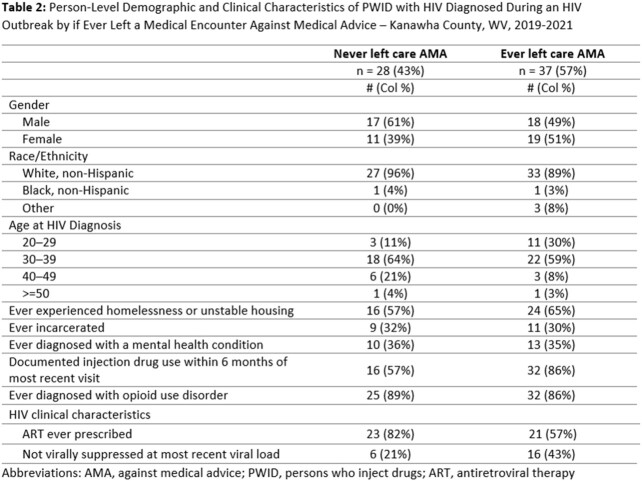

**Conclusion:**

The high frequency of leaving encounters AMA highlights serious gaps in treatment services and barriers to care for PWID associated with this outbreak. Challenges related to complex medical and social needs, including recent IDU, and prior negative experiences in healthcare facilities may have contributed to PWID leaving medical encounters AMA. Patient-centered care that addresses these challenges could improve patient experiences and health outcomes among PWID.

**Disclosures:**

**All Authors**: No reported disclosures

